# Visualization of Lokiarchaeia and Heimdallarchaeia (Asgardarchaeota) by Fluorescence *In Situ* Hybridization and Catalyzed Reporter Deposition (CARD-FISH)

**DOI:** 10.1128/mSphere.00686-20

**Published:** 2020-07-29

**Authors:** Michaela M. Salcher, Adrian-Ştefan Andrei, Paul-Adrian Bulzu, Zsolt G. Keresztes, Horia L. Banciu, Rohit Ghai

**Affiliations:** a Department of Aquatic Microbial Ecology, Institute of Hydrobiology, Biology Centre of the Czech Academy of Sciences, České Budějovice, Czech Republic; b Limnological Station, Institute of Plant and Microbial Biology, University of Zurich, Kilchberg, Switzerland; c Department of Molecular Biology and Biotechnology, Faculty of Biology and Geology, Babeş-Bolyai University, Cluj-Napoca, Romania; d Molecular Biology Center, Institute for Interdisciplinary Research in Bio-Nano-Sciences, Babeş-Bolyai University, Cluj-Napoca, Romania; University of Wisconsin—Madison

**Keywords:** Asgardarchaeota, Heimdallarchaeia, Lokiarchaeia, CARD-FISH, morphology

## Abstract

Metagenome-assembled genomes (MAGs) of Asgardarchaeota have been recovered from a variety of habitats, broadening their environmental distribution and providing access to the genetic makeup of this archaeal lineage. The recent success in cultivating the first representative of Lokiarchaeia was a breakthrough in science at large and gave rise to new hypotheses about the evolution of eukaryotes. Despite their singular phylogenetic position at the base of the eukaryotic tree of life, the morphology of these bewildering organisms remains a mystery, except for the report of an unusual morphology with long, branching protrusions of the cultivated Lokiarchaeion strain “*Candidatus* Prometheoarchaeum syntrophicum” MK-D1. In order to visualize this elusive group, we applied a combination of fluorescence *in situ* hybridization and catalyzed reporter deposition (CARD-FISH) and epifluorescence microscopy on coastal hypersaline sediment samples, using specifically designed CARD-FISH probes for Heimdallarchaeia and Lokiarchaeia lineages, and provide the first visual evidence for Heimdallarchaeia and new images of a lineage of Lokiarchaeia that is different from the cultured representative. Here, we show that while Heimdallarchaeia are characterized by a uniform cellular morphology typified by a centralized DNA localization, Lokiarchaeia display a plethora of shapes and sizes that likely reflect their broad phylogenetic diversity and ecological distribution.

**IMPORTANCE** Asgardarchaeota are considered to be the closest relatives to modern eukaryotes. These enigmatic microbes have been mainly studied using metagenome-assembled genomes (MAGs). Only very recently, a first member of Lokiarchaeia was isolated and characterized in detail; it featured a striking morphology with long, branching protrusions. In order to visualize additional members of the phylum Asgardarchaeota, we applied a fluorescence *in situ* hybridization technique and epifluorescence microscopy on coastal hypersaline sediment samples, using specifically designed probes for Heimdallarchaeia and Lokiarchaeia lineages. We provide the first visual evidence for Heimdallarchaeia that are characterized by a uniform cellular morphology typified by an apparently centralized DNA localization. Further, we provide new images of a lineage of Lokiarchaeia that is different from the cultured representative and with multiple morphologies, ranging from small ovoid cells to long filaments. This diversity in observed cell shapes is likely owing to the large phylogenetic diversity within Asgardarchaeota, the vast majority of which remain uncultured.

## Abstract (precis)

Asgardarchaeota are considered to be the closest relatives to modern eukaryotes. These enigmatic microbes have been mainly studied using metagenome-assembled genomes (MAGs). Only very recently, a first member of Lokiarchaeia was isolated and characterized in detail; it featured a striking morphology with long, branching protrusions. In order to visualize additional members of the phylum Asgardarchaeota, we applied a fluorescence *in situ* hybridization technique and epifluorescence microscopy on coastal hypersaline sediment samples, using specifically designed probes for Heimdallarchaeia and Lokiarchaeia lineages. We provide the first visual evidence for Heimdallarchaeia that are characterized by a uniform cellular morphology typified by an apparently centralized DNA localization. Further, we provide new images of a lineage of Lokiarchaeia that is different from the cultured representative and with multiple morphologies, ranging from small ovoid cells to long filaments. This diversity in observed cell shapes is likely owing to the large phylogenetic diversity within Asgardarchaeota, the vast majority of which remain uncultured.

## INTRODUCTION

The discovery of the Asgardarchaeota not only revealed the closest archaeal lineage to the eukaryotic ancestor but even more unexpectedly demonstrated that the descendants of the same archaeal lineage are still with us today (1–4). Recent analyses resulted in a robust support for a two-domain tree of life, with Heimdallarchaeia being identified as the best candidates for the closest archaeal relatives to eukaryotes (5–7). All Asgardarchaeota possess genes that are homologous to eukaryotic genes involved in ubiquitin and cytoskeleton formation, vesicle/membrane trafficking or remodeling, and phagocytosis (3, 4, 6). These archaea are highly diverse, and sequences of the Asgardarchaeota superphylum have been reported from a wide range of habitats including marine, brackish, and freshwater sediments; hot springs; marine pelagic zones; and saline microbial mats (3, 4, 6, 8–12). Asgardarchaeota exhibit a high metabolic versatility, including carbon fixation, fermentation, halogenated organic compound metabolism, hydrocarbon oxidation, and variable hydrogen consumption and production (7–9, 13). While all so far published Thor-, Odin-, Hel-, and Lokiarchaeia have anaerobic lifestyles (4, 7–9, 12), several Heimdallarchaeia appear capable of facultative aerobic metabolism, also possessing at least three types of light-activated rhodopsins (6).

The recent success in cultivating the first representative of Lokiarchaeia (“*Candidatus* Prometheoarchaeum syntrophicum” MK-D1 [14]) was a breakthrough in an ongoing discussion about the reliability of metagenome-assembled genomes (MAGs) of Asgardarchaeota (15–18), as it proved the existence of these enigmatic microbes. “*Ca*. Prometheoarchaeum syntrophicum” MK-D1, a member of Lokiarchaeia, was enriched in a decade-long cocultivation with a sulfate-reducing and a methanogenic partner that supply amino acids and peptides for syntrophic growth. This anaerobic archaeon displayed extremely slow growth and an unusual variable morphology consisting mainly of small cocci (550 nm in diameter) with long, branching protrusions (variable lengths, 80 to 100 nm in diameter) (14). Although no visible organelle-like structures and no phagocytic behavior have been reported from this strain, a new hypothetical model for eukaryogenesis has been proposed (entangle-engulf-endogenize model) by Imachi and coworkers (14).

Despite the overall growing appreciation of these remarkable microbes, a pressing concern is that not a single member of Heimdallarchaeia has yet been seen and only one cultivated strain of Lokiarchaeia with unusual morphology has been described so far. Motivated by our recent recovery of one of the largest collections of Asgardarchaeota MAGs from brackish sediments of Lakes Amara and Tekirghiol (Romania) (6), we chose to tackle this issue by using fluorescence *in situ* hybridization and catalyzed reporter deposition (CARD-FISH) and epifluorescence microscopy techniques that have been previously successfully used for visualization of a wide array of microbes (19–22).

(This paper has been released as a preprint at bioRxiv [23].)

## RESULTS AND DISCUSSION

**FIG 1:**
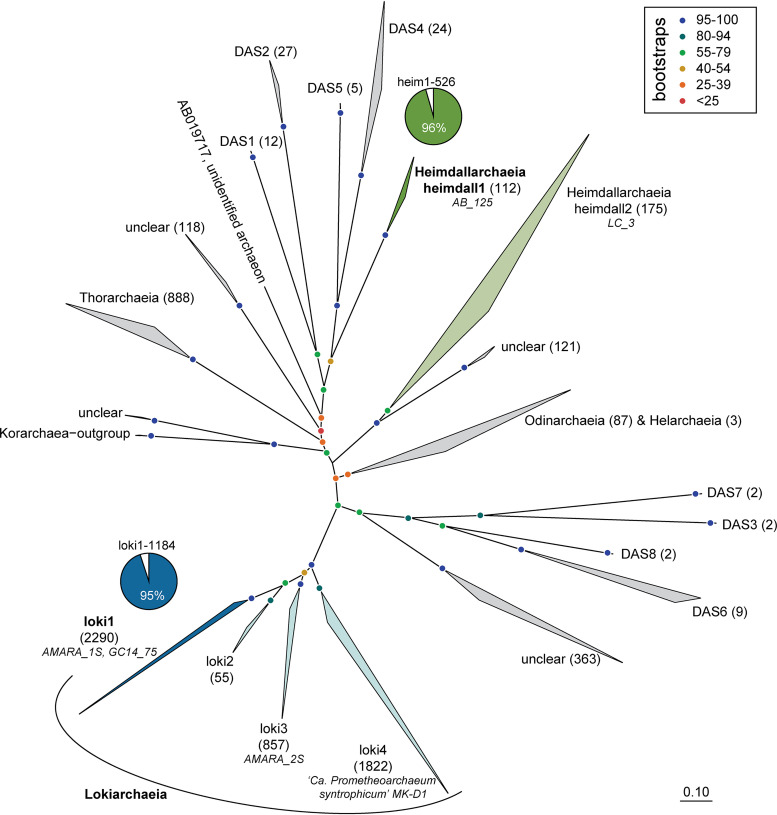
RAxML tree (GTR-gamma model, 100 bootstraps) of 16S rRNA genes of Asgardarchaeota. The number of sequences for collapsed branches is given in parentheses, and 16S rRNA gene sequences of MAGs and cultures affiliated with Lokiarchaeia and Heimdallarchaeia are given in italics. The scale bar at the bottom indicates 10% sequence divergence. Fractions of hits of probes loki1-1184 and heim1-526 are displayed as pie charts. DAS, domain archaeal sequences (28); unclear, lineages of unclear affiliation.

All attempts to construct a general probe targeting all Asgardarchaeota sequences (*n* = 6,977) failed, likely due to the high diversity of this superphylum (Fig. 1). The impossibility of designing such broad-range probes is not surprising, as similar difficulties have been reported for other diverse prokaryotic groups (e.g., *Proteobacteria* [21]). Even the widely used “general” archaeal probe ARCH915 (24) is unspecific in this regard, overlapping only partially with Asgardarchaeota (85% coverage; SILVA TestProbe against SSURef_138 [25]). This “general” archaeal probe covers 89% of all archaea and 95% Lokiarchaeia but fails to detect most Heimdallarchaeia (only 2% targeted) and Odinarchaeia (17% targeted) and is thus not sufficiently reliable to detect Asgardarchaeota. Another set of probes, originally designed for Archaea of the marine benthic group B (MBG-B) that later turned out to be members of Lokiarchaeia (26), covers 69 to 93% of all Lokiarchaeia but has a large number of outgroup hits (99 to 142 sequences affiliated with Crenarchaeota, Micrarchaeia, and Aenigmarchaeota; i.e., 19 to 24% of total hits are not affiliated with Asgardarchaeota [see Table S1 in the supplemental material]). While these probes do target many Lokiarchaeia, with such high levels of uncertainty these probes cannot be considered very specific.

10.1128/mSphere.00686-20.9 TABLE S1 Details of four probes targeting Archaea of the marine benthic group B (MBG-B), designed by Knittel et al. (K. Knittel, T. Lösekann, A. Boetius, R. Kort, and R. Amann, Appl Environ Microbiol 71:467–479, 2005, https://doi.org/10.1128/AEM.71.1.467-479.2005). Download Table S1, DOCX file, 0.01 MB. Copyright © 2020 Salcher et al. 2020 Salcher et al. This content is distributed under the terms of the Creative Commons Attribution 4.0 International license.

Consequently, we decided to design two specific probes for lineages within Heimdall- and Lokiarchaeia. Probe loki1-1184 targets 95% of sequences affiliated with lineage loki1, the largest of the four branches of Lokiarchaeia (2,290 sequences in total) including MAGs AMARA_1S (SDNY00000000) from Lake Amara (6) and GC14_75 (JYIM01000321) from Loki’s Castle (3, 4). This lineage was previously described as Deep Sea Archaeal Group (DSAG)-Gamma; (27) or Lokiarchaeota-Group3 (13), and members of this group seem to have the broadest environmental distribution and pH tolerance (13). Lineage loki2 (also described as DSAG-Alpha [27] or Lokiarchaeota-Group1 [13]) does not contain genomes with 16S rRNA gene sequences and represents a minor group containing 55 sequences. Lineage loki3 (also described as DSAG-Beta2 [27] or Lokiarchaeota-Group2B [13]), on the other hand, contains another MAG (AMARA_2S; SDNS00000000) gained from Lake Amara (6). Finally, lineage loki4 (also known as DSAG-Beta1 [27] or Lokiarchaeota-Group2A [13]) contains the first cultivated representative of Asgardarchaeota, “*Ca*. Prometheoarchaeum syntrophicum” MK-D1 (CP042905) (14). Our probe loki1-1184 targets exclusively members of lineage loki1 (95% coverage) but none of the other three lineages (Fig. 1 and Table 1; see also Table S2).

**TABLE 1:** Details of the newly designed probes

Probe name	Targeted lineage (MAGs)	Probe sequence (5′–3′)	% coverage (no. of hits)	Outgroup hits	% formamide	Avg length (μm) (±SD)	Avg width (μm) (±SD)	No. of cells measured
loki1-1184	Lokiarchaeia lineage loki1 (AMARA_1S, GC14_75)	GACCTGCCTTTGCCCGC	95 (2,175)	None	55	3.670 ± 4.10	1.423 ± 0.50	37
heim1-526	Heimdallarchaeia lineage heimdall1 (Heimdall_AB_125)	CACTCGCAGAGCTGGTT TTACCG	95.5 (107)	1 DAS5 (FN820420)	40	2.005 ± 0.47	1.422 ± 0.38	23

10.1128/mSphere.00686-20.10 TABLE S2 16S rRNA gene sequences targeted by the newly designed probes and competitors. Putative chimera (pintail values <50) are marked in red, and sequences with changed taxonomy after alignment optimization and RAxML tree construction are marked in green. Download Table S2, XLSX file, 0.1 MB. Copyright © 2020 Salcher et al. 2020 Salcher et al. This content is distributed under the terms of the Creative Commons Attribution 4.0 International license.

Probe heim1-526 targets 95.5% of a specific lineage of Heimdallarchaeia that we designated heimdall1 (targeting 107 sequences [Fig. 1, Table 1, and Table S2]). This lineage contains one MAG (AB_125, MEHH01000036) (4) and is closely related to lineages DAS1 (domain archaeal sequences), DAS2, DAS4, and DAS5 (28), while a second branch of Heimdallarchaeia (heimdall2, containing MAG LC_3 [MDVS00000000] [4]) appears only distantly related. The polyphyletic nature of Heimdallarchaeia in 16S rRNA gene trees has been noted before (13, 28). Most published MAGs of Heimdallarchaeia are only distantly related to each other with average nucleotide identities (ANIs) (29) of <65%, with two exceptions: one group of MAGs (MAGs AB_125 [MEHH00000000], AMARA_4 [SDNT00000000], and E29_bin46 [SOIU00000000]) (4, 6) affiliated with lineage heimdall1 has slightly higher ANI values (70 to 78%), and another group of MAGs (B5_G9 [QMYX00000000], B33_G2 [QMYY00000000], and B18_G1 [QMYZ00000000]; all gained from different sites of the Guaymas Basin, Gulf of California) has almost identical genomes (ANI >99%).

During *in silico* testing of the designed probes for specificity and group coverage, we identified several sequences that behaved aberrantly, i.e., could theoretically be regarded as outgroup hits for probe loki1-1184 (GU363076 and EU731577, Table S2). However, these were discarded after closer examination because of pintail values of 20 and 0, respectively, indicating a chimeric origin (30). Pintail values are measures of chimeric nature for rRNA sequences in the databases, a value closer or equal to 100 indicating a nonchimeric sequence (30). Additional outgroup candidates for probe loki1-1184 (AY133348, JQ817340, and KU351219, Table S2) were initially located within Heimdallarchaeia in the guide tree provided by SILVA but turned out to belong to Lokiarchaeia after alignment optimizations and RAxML tree reconstruction. To be absolutely certain to avoid false-positive signals, i.e., to target no other organisms, we designed a set of competitor oligonucleotides that bind specifically to those rRNA sequences that have a single mismatch with our probes (31). We designed three distinct competitor probes for heimdall1 and two for loki1 (Table 2). Mismatch and competitor analyses using the online tool mathFISH (32) resulted in 0 to 1% hybridization efficiency for nontarget hits with 1 mismatch with use of competitors (Fig. S1 and S2). Each competitor was used in the same concentrations as the CARD-FISH probes in order to prevent nonspecific binding. The usage of specific probes together with competitors has been previously shown to work very well for visualizing cell morphology and enumeration and was applied numerous times (19, 20, 22, 33, 34).

**TABLE 2:** Details of the newly designed competitors

Competitor name	Description	Sequence	Taxonomy (no.) of target hits
loki1-1184-C1	Competitor 1 for loki1-1184	GACCTGCCGTTGCCCGC	Bathyarchaeia (37), Archaeoglobi (53), putative chimera (1)
loki1-1184-C2	Competitor 2 for loki1-1184	GACATGCCTTTGCCCGC	Bathyarchaeia (2)
heim-526-C1	Competitor 1 for heim1-526	CACTCGRAGAGCTGGTTTTACCG	Bathyarchaeia (31), Odinarchaeia (42), Lokiarchaeia (1), unclassified Asgardarchaeota (1), Thorarchaeia (4), putative chimera (10)
heim-526-C2	Competitor 2 for heim1-526	CACTCGCAGAGCTGGTATTACCG	Bathyarchaeia (2)
heim-526-C3	Competitor 3 for heim1-526	CACTCGCGGAGCTGGTTTTACCG	Uncultured Archaea (5)

10.1128/mSphere.00686-20.1 FIG S1 Stringent conditions for probe loki1-1184 and mismatch discrimination by the use of two competitors. (a) Formamide curve for a target hit (AB525489) and two nontarget hits with one mismatch to probe loki1-1184 (AB274307 and FJ902705). Optimal stringent hybridization conditions for the probe were identified at 55% formamide concentrations (FA%). (b) Competitor analysis for competitor 1. (c) Competitor analysis for competitor 2. Please note that the use of both competitors results in 0% hybridization efficiency for nontarget hits. Formamide curves, mismatches, and competitor analyses were computed with mathFISH (http://mathfish.cee.wisc.edu/index.html). Download FIG S1, TIF file, 2.5 MB. Copyright © 2020 Salcher et al. 2020 Salcher et al. This content is distributed under the terms of the Creative Commons Attribution 4.0 International license.

10.1128/mSphere.00686-20.2 FIG S2 Stringent conditions for probe heim1-526 and mismatch discrimination by the use of three competitors. (a) Formamide curve for a target hit (MEHH01000036) and four nontarget hits with one mismatch to probe heim1-526 (AB019718, AB237760, GU553614, and DQ228522). Optimal stringent hybridization conditions for the probe were identified at 40% formamide concentrations (FA%). (b) Competitor analysis for competitor 1 that has a wobble at position 7 (R = G or A) which was tested for two sequences with both variants. (c) Competitor analysis for competitor 2. (d) Competitor analysis for competitor 3. Please note that the use of all three competitors results in 1 to 0% hybridization efficiency for nontarget hits. Formamide curves, mismatches, and competitor analyses were computed with mathFISH (http://mathfish.cee.wisc.edu/index.html). Download FIG S2, TIF file, 2.5 MB. Copyright © 2020 Salcher et al. 2020 Salcher et al. This content is distributed under the terms of the Creative Commons Attribution 4.0 International license.

### Occurrence and cell shapes of Loki- and Heimdallarchaeia in sediment samples.

**FIG 2:**
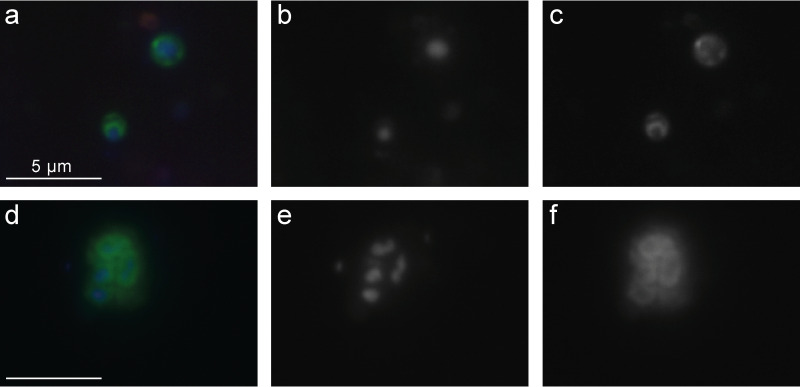
CARD-FISH imaging of Heimdallarchaeia hybridized with probe heim1-526. The left panels (a and d) display overlay images of probe signal (green), DAPI staining (blue), and autofluorescence (red); the middle panels (b and e) show DAPI staining of DNA; the right panels (c and f) show CARD-FISH staining of proteins. Individual microphotographs of autofluorescent objects are not displayed because of low intensities and no interference with probe signals (see Fig. S5). The scale bar (5 μm) in the left images applies to all microphotographs. The displayed images were recorded from samples originating from the top sediment layer (0 to 10 cm) of Lake Tekirghiol; additional images of Heimdallarchaeia from other sediment samples can be found in Fig. S3.

**FIG 3:**
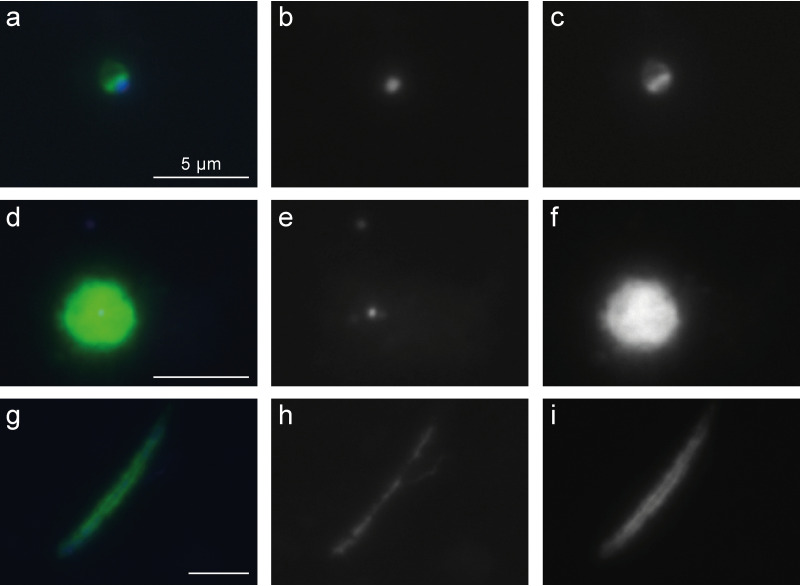
CARD-FISH imaging of Lokiarchaeia hybridized with probe loki1-1184. Three different morphotypes are displayed: small to medium-sized ovoid cells detectable in Lake Tekirghiol sediment layers 0 to 10, 10 to 20, and 20 to 30 cm and the top-0- to 10-cm sediment in Lake Amara, respectively (a to c); a large round cell detected only in the top 10 cm of Lake Amara (d to f); and large filamentous cells detected in Lake Tekirghiol sediment layer 20 to 30 cm and the top 0 to 10 cm of Lake Amara (g to i). The left panels (a, d, and g) display overlay images of probe signal (green), DAPI staining (blue), and autofluorescence (red); the middle panels (b, e, and h) show DAPI staining of DNA; the right panels (c, f, and i) show CARD-FISH staining of proteins. Individual microphotographs of autofluorescent objects are not displayed because of low intensities and no interference with probe signals (see Fig. S5). The scale bar (5 μm) in the left images applies to all microphotographs. The displayed images were all recorded from the top-10-cm sediment of Lake Amara; additional images of Lokiarchaeia from different sediment samples can be found in Fig. S4 and S5.

**FIG 4:**
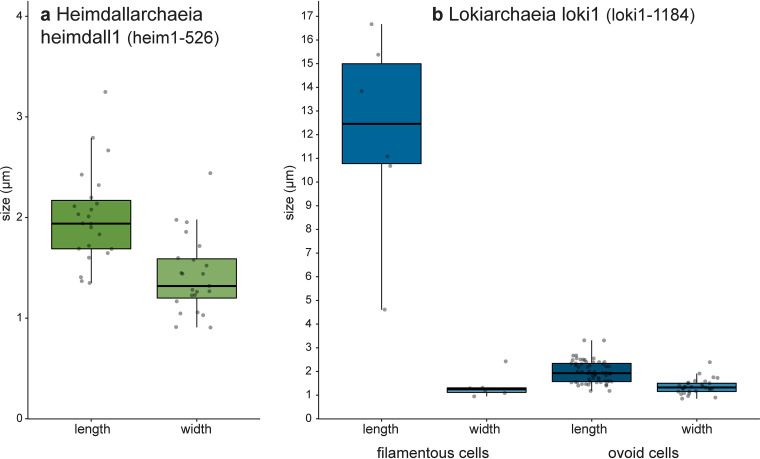
Cell sizes (lengths and widths) of Heimdallarchaeia lineage heimdall1 (number of cells used for sizing *n* = 23) (a) and two different morphotypes of Lokiarchaeia lineage loki1 (filaments, *n* = 6; small to medium-sized ovoid cells, *n* = 30) (b). Boxplots display median (solid line), 25th and 75th percentiles (boxes), and 5th and 95th percentiles (whiskers) as well as all individual values (gray dots).

We applied the probes in sediment samples from Lake Amara and Lake Tekirghiol, sites from where recently several Asgardarchaeota genomes were recovered by metagenomics (6). Both lineages of Loki- and Heimdallarchaeia were rare in sediment samples taken from Lake Amara and Lake Tekirghiol during the April 2018 sampling campaign (detailed in reference 6) and appeared completely absent below depths of 40 cm. At both sites, the water column is oxic due to mixing (water depth at sediment sampling locations: 0.8 m in Tekirghiol and ∼2 m in Amara). Moreover, the top layers of both sampled sediments are inferred to be microoxic niches owing to the presence of multiple aerobic metabolic pathways in Heimdallarchaeia MAGs that were found here (6). All observed Heimdallarchaeia were similar in cell size (2.0 ± 0.5 μm in length by 1.4 ± 0.4 μm in width, *n* = 23) and of conspicuous shape with DNA condensed (0.8 ± 0.2 by 0.5 ± 0.2 μm) at the center of the cells (Fig. 2, Fig. S3, and Fig. S6a to d; see also Fig. 4a), which is rather atypical for prokaryotes. In contrast, Lokiarchaeia presented diverse shapes and sizes, and we could distinguish at least two distinct morphotypes. The most common Lokiarchaeia were small to medium-sized, ovoid cells (2.0 ± 0.5 by 1.4 ± 0.3 μm, *n* = 30 [Fig. 3a to c, Fig. 4b, and Fig. S4]) that were found at different sediment depths in Lake Tekirghiol (0 to 10 cm, 10 to 20 cm, and 20 to 30 cm) and in the top-10-cm sample from Lake Amara. A single large round cell (3.8 by 3.6 μm, Fig. 2d to f) with bright fluorescence signal and condensed DNA at the center was detected in Lake Amara; however, as only one individual cell was observed, this shape cannot be considered representative of this lineage. On the other hand, several large rods/filaments (12.0 ± 4.3 by 1.4 ± 0.5 μm, *n* = 6 [Fig. 3g to i, Fig. 4b, Fig. S5, and Fig. S6e to h) with filamentous, condensed DNA (10.2 ± 4.8 by 0.6 ± 0.1 μm) were present at 30- to 40-cm sediment depth in Lake Tekirghiol and in 0- to 10-cm depth in Lake Amara. The variety of Lokiarchaeia morphologies most likely reflects the higher sampling of the phylogenetic diversity within this phylum. Our probe loki1-1184 targets a specific branch of Lokiarchaeia (loki1, Fig. 1) that includes MAGs AMARA_1 recovered from Lake Amara (6) and GC14_75 recovered from Loki’s Castle in the Arctic Ocean (3). The recently described cultivated representative of Lokiarchaeia “*Candidatus* Prometheoarchaeum syntrophicum” MK-D1 is a member of lineage loki4 (Fig. 1) (14) and was reported to be morphologically complex with long and often branching protrusions (14). We did not record similar cell shapes in any of our analyzed samples (Fig. 3 and Fig. S4 to S6), which is not surprising as “*Ca*. Prometheoarchaeum syntrophicum” is only very distantly related to lineage loki1 (ANI values <63%) and our probe has 3 mismatches with the 16S rRNA gene sequence of this organism. Moreover, all cells visualized by our probe were much larger in size (Fig. 4) than “*Ca*. Prometheoarchaeum syntrophicum” MK-D1 (550 nm in diameter). The reported protrusions in the cultured representative, given their width of 80 to 100 nm, are likely beyond the resolving power of normal epifluorescence microscopy at magnifications of ×1,000, even if they would be full of ribosomes and targeted by CARD-FISH. Moreover, it is not expected that a phylum as diverse as Lokiarchaeia presents only a single morphotype.

10.1128/mSphere.00686-20.3 FIG S3 CARD-FISH images of Heimdallarchaeia lineage heim1 (probe heim1-526). The left panels display overlay images of probe signal (green), DAPI staining (blue), and autofluorescence (red); the middle panels show DAPI staining of DNA; the right panels show CARD-FISH staining of proteins. Individual microphotographs of autofluorescent objects are not displayed because of low intensities and no interference with probe signals. Download FIG S3, TIF file, 1.9 MB. Copyright © 2020 Salcher et al. 2020 Salcher et al. This content is distributed under the terms of the Creative Commons Attribution 4.0 International license.

10.1128/mSphere.00686-20.4 FIG S4 CARD-FISH imaging of a distinct morphotype with medium-sized ovoid cells of Lokiarchaeia lineage loki1 (probe loki1-1184). The left panel displays overlay images of probe signal (green), DAPI staining (blue), and autofluorescence (red); the middle panel shows DAPI staining of DNA; the right panel shows CARD-FISH staining of proteins. Individual microphotographs of autofluorescent objects are not displayed because of low intensities and no interference with probe signals. The scale bar (5 μm) in the left images applies to all microphotographs. Download FIG S4, TIF file, 2.0 MB. Copyright © 2020 Salcher et al. 2020 Salcher et al. This content is distributed under the terms of the Creative Commons Attribution 4.0 International license.

10.1128/mSphere.00686-20.5 FIG S5 CARD-FISH imaging of the filamentous morphotype of Lokiarchaeia lineage loki1 (probe loki1-1184). The left panel displays overlay images of probe signal (green), DAPI staining (blue), and autofluorescence (red); the middle panel shows DAPI staining of DNA; the right panel shows CARD-FISH staining of proteins. Individual microphotographs of autofluorescent objects are not displayed because of low intensities and no interference with probe signals. The scale bar (5 μm) in the left images applies to all microphotographs. Download FIG S5, TIF file, 1.2 MB. Copyright © 2020 Salcher et al. 2020 Salcher et al. This content is distributed under the terms of the Creative Commons Attribution 4.0 International license.

10.1128/mSphere.00686-20.6 FIG S6 Images of Heimdallarchaeia lineage heim1 (a to d) and Lokiarchaeia lineage loki1 (e to h) at ×400 magnification with insets taken at ×1,000 magnification. (a and e) Overlay images of probe signal (green), DAPI staining (blue), and autofluorescence (red); (b and f) DAPI staining; (c and g) CARD-FISH staining; (d and h) autofluorescence. Images were recorded at fixed exposure times (70 or 100 ms for DAPI, 100 or 200 ms for CARD-FISH, and 100 or 400 ms for autofluorescence for ×400 or ×1,000 magnification, respectively). Download FIG S6, TIF file, 2.3 MB. Copyright © 2020 Salcher et al. 2020 Salcher et al. This content is distributed under the terms of the Creative Commons Attribution 4.0 International license.

Precise quantification of both lineages in different sediment layers was hampered by the very low abundances in the analyzed samples, which correspond well to low recoveries of Asgardarchaeota 16S rRNA reads from metagenomes (Fig. S7) (4). Consequently, it is difficult to draw firm conclusions on sediment depth preferences or rule out additional morphotypes of Lokiarchaeia.

10.1128/mSphere.00686-20.7 FIG S7 Abundance estimation of recovered 16S rRNA reads affiliated with Heimdallarchaeia (a) and Lokiarchaeia (b) from metagenomes from Loki’s Castle, Lake Tekirghiol, and Lake Amara. Shotgun metagenomes from Amara (SRA7615342) and Tekirghiol (SRA7614767) lakes, as well as the published SRX684858 from Loki’s Castle, were subsampled to 20 million sequences. Each subset was queried for putative RNA sequences against the nonredundant SILVA SSURef_NR99_132 database, which was clustered at 85% sequence identity. Identified putative 16S rRNA sequences (E value <1e−5) were screened using SSU-ALIGN. Resulting bona fide 16S rRNA sequences were compared by blastn (E value <1e−5) against the curated SILVA SSURef_NR99_132 database. Matches with identity of ≥80% and alignment length of ≥90 bp were considered for downstream analyses. Sequences assigned to Loki- and Heimdallarchaeia were used to calculate abundances for these taxa in their originating environments. Download FIG S7, TIF file, 0.3 MB. Copyright © 2020 Salcher et al. 2020 Salcher et al. This content is distributed under the terms of the Creative Commons Attribution 4.0 International license.

During microscopic inspections, we carefully checked for potential nonspecific or autofluorescent signals at wavelengths not interfering with the probe signal and found no overlap for any of the inspected cells. A set of negative controls was conducted to rule out false-positive signals due to unspecific binding of dye or nucleic acid components of probes by using a nonspecific probe (NON338 [35]). To avoid false-positive signals from cellular peroxidases, we performed additional control experiments including the CARD reaction only (without probes). All these control treatments resulted in low, unspecific background signals (comparable to the local background in samples with probes for Asgardarchaeota) but no obvious staining of cells (Fig. S8). Additionally, we performed CARD-FISH with the general archaeal probe Arch915 to make sure that CARD-FISH works well for our samples. Further evidence of specificity was seen in all cells hybridized with the Heimdallarchaeia probe; both the shapes and staining patterns coupled to 4′,6-diamidino-2-phenylindole (DAPI) were remarkably consistent.

10.1128/mSphere.00686-20.8 FIG S8 Control treatments with nonsense probe NON338 (a to d), the general archaeal probe Arch915 (e to h), and the CARD reaction only without using a probe (i to l). (a, e, and i) Overlay images of probe signal (green), DAPI staining (blue), and autofluorescence (red); (b, f, and j) DAPI staining; (c, g, and k) CARD-FISH or CARD-only staining; (d, h, and l) autofluorescence. Images were recorded at fixed exposure times (70 ms for DAPI, 100 ms for CARD-FISH, and 100 ms for autofluorescence). Download FIG S8, TIF file, 2.7 MB. Copyright © 2020 Salcher et al. 2020 Salcher et al. This content is distributed under the terms of the Creative Commons Attribution 4.0 International license.

While tempting, in the absence of strong supporting evidence it would be too premature to conclude whether the condensed DNA, particularly in Heimdallarchaeia cells, is indicative of a protonucleus. Microscopic images of bacterial cells with apparently eukaryotic features have been misinterpreted before, e.g., in the case of the phylum *Planctomycetes* (36). Similarly, no obvious cell compartmentalization was reported in the ultrastructure of the recently isolated strain “*Ca*. Prometheoarchaeum syntrophicum” (14). The availability of additional enrichment/pure cultures might be necessary to firmly resolve these outstanding issues.

## MATERIALS AND METHODS

### Phylogenetic analyses and probe design.

In order to design specific probes for a morphological characterization of Asgardarchaeota, we manually optimized the alignment of all 16S rRNA gene sequences classified as Asgardarchaeota in ARB (37) using SILVA database SSURef_NR99_132 (25) amended with 6,647 near-full-length sequences that were originally not included in this database (28). An RAxML tree (GTR-gamma model, 100 bootstraps [38]) was constructed for all high-quality near-full-length sequences (Fig. 1). Specific probes for Heimdallarchaeia lineage heimdall1 and Lokiarchaeia lineage loki1 and a set of competitor probes (31) were designed using the tools Probe_Design and Probe_Match in ARB (37). All probes were tested *in silico* for specificity and coverage within ARB and online using the TestProbe function of SILVA (25). An *in silico* test of the probes and competitors was carried out with the online tool mathFISH (32) using the formamide curve generator (39), mismatch analysis (40), and competitor analysis (41) functions with randomly chosen almost-full-length nontarget hits with 1 mismatch each (see Fig. S1 and S2 in the supplemental material). The resulting optimal formamide concentrations of 55% and 40% for probes loki1-1184 and heim1-526, respectively, were verified in the laboratory using different formamide concentrations (45, 50, 55, and 60% and 30, 35, 40, and 45% formamide for loki1-1184 and heim1-526, respectively) in the hybridization buffer until stringent conditions were achieved (Table 1).

### Sediment sampling.

We tested these probes in sediment samples from two sites from where recently several Asgardarchaeota genomes were recovered by metagenomics (6): Lake Amara (44°36.30650 N, 27°19.52950 E; 32 m above sea level [a.s.l.]; 1.3-km^2^ area; maximum depth 6 m) and Lake Tekirghiol (44°03.19017 N, 28°36.19083 E; 0.8 m a.s.l.; 11.6-km^2^ area; maximum depth 9 m, salinity 6%) are naturally formed shallow lakes in southeastern Romania that harbor large deposits of organic-rich sediments (42). Sediment sampling was performed using a custom mud corer on 22 and 23 April 2018. Five sediment layers (0 to 50 cm, in 10-cm ranges) were sampled in Lake Tekirghiol, and the top 10 cm was sampled in Lake Amara. Additional details regarding sampling procedures, origin of lakes, chemical analyses of sediments, and rRNA-based abundance estimates of Loki-, Heimdall- and Odinarcheia lineages were presented in the work of Bulzu et al. (6) (Methods section, Table S1, Table S9, and Fig. S7).

### CARD-FISH.

Samples were fixed with formaldehyde for 1 h and washed three times with 1× phosphate-buffered saline (PBS), with centrifugation at 16,000 × *g* for 5 min between washes and a final resuspension in a 1:1 mixture of PBS and ethanol. A treatment of sonication (20 s, minimum power) on ice, vortexing, and centrifugation to detach cells from sediment particles was applied (43), and aliquots diluted with PBS (1:10 dilution) were filtered onto white polycarbonate filters (0.2-μm pore size; Millipore). Filters were treated with permeabilization steps with lysozyme (10 mg/ml of lysozyme, 50 mM EDTA, and 0.1 M Tris-HCl, 30 min, 37°C) and achromopeptidase (60 U, 1 mM NaCl, 1 mM Tris-HCl, 25 min, 37°C) and an inactivation step for cellular peroxidases with 0.15% H_2_O_2_ (in methanol, 30 min, room temperature) (43). Fluorescence *in situ* hybridization followed by catalyzed reporter deposition (CARD-FISH) was conducted as previously described with fluorescein-labeled tyramides. The following horseradish peroxidase (HRP)-labeled probes were used for hybridization for 2 h at 35°C: loki1-1184, heim1-526 (Table 1 shows details), and NON338 (35), and Arch915 (24) as negative controls for unspecific binding of a general nontarget probe and general target probe for archaea, respectively. Another negative control for unspecific binding of fluorescein and cellular peroxidases was done by carrying out the CARD reaction only, i.e., FISH was done without adding a probe to the hybridization reaction mixture. All filters were counterstained with DAPI and inspected by epifluorescence microscopy (Zeiss Imager.M1) with filter sets for DAPI (filter set 01: BP [band pass] 365/12, FT [farb teiler] 395, LP [long pass] 397), fluorescein (filter set 10: BP 450 to 490, FT 510, BP 515 to 565), and autofluorescence (filter set 15: BP 546/12, FT 580, LP 590). Micrographs of CARD-FISH-stained cells were recorded with a highly sensitive charge-coupled device (CCD) camera (Vosskühler) at fixed exposure times (70 and 100 ms for DAPI, 100 and 200 ms for CARD-FISH, and 100 and 400 ms for autofluorescence for magnifications of ×400 and ×1,000, respectively), and cell sizes were estimated with the software LUCIA (Laboratory Imaging, Prague, Czech Republic).

### Abundance estimates of recovered 16S rRNA reads from metagenomes.

Shotgun metagenomes from Lakes Amara (SRA7615342) and Tekirghiol (SRA7614767), as well as the published SRX684858 sequence from Loki’s Castle, were subsampled to 20 million sequences. Each subset was queried for putative RNA sequences against the nonredundant SILVA SSURef_NR99_132 database, which was clustered at 85% sequence identity. Identified putative 16S rRNA sequences (E value <1e−5) were screened using SSU-ALIGN. Resulting bona fide 16S rRNA sequences were compared by blastn (E value <1e−5) against the curated SILVA SSURef_NR99_132 database. Matches with identity of ≥80% and alignment length of ≥90 bp were considered for downstream analyses. Sequences assigned to Loki- and Heimdallarchaeia were used to calculate abundances for these taxa in their originating environments.
