# Supplementary material for: Visualization of Lokiarchaeia and Heimdallarchaeia (Asgardarchaeota) by Fluorescence In Situ Hybridization and Catalyzed Reporter Deposition (CARD-FISH)
Source: mSphere. 2020 Jul 29;5(4):e00686-20. doi: 10.1128/mSphere.00686-20 (PMC7392546; doi:10.1128/mSphere.00686-20)
Supplement: TABLE S1 [file mSphere.00686-20-st001.docx]

Table S1: Details of four probes targeting Archaea of the Marine benthic group B (MBG-B), designed by Knittel et al. 2005.

| **Probe name** | **Target group** | **# total hits** | **# hits Asgard-archaeota** | **# hits Loki-archaeia** | **# hits other Asgard-archaeota** | **# outgroup hits** | **Taxonomy of outgroup hits** | **coverage Asgard-archaeota (%)** | **coverage Lokiarchaeia (%)** | **outgroup hits (% of total hits)** |
| --- | --- | --- | --- | --- | --- | --- | --- | --- | --- | --- |
| MBGB-280 | Lokiarchaeia (MBG-B) | 528 | 427 | 425 | 1 Odin | 101 | 100 Crenarcheaeota, 1 Micrarchaeia | 66.1 | 82.8 | 19.1 |
| MBGB-335 | Lokiarchaeia (MBG-B) | 551 | 447 | 434 | 13 Odin | 104 | 101 Crenarchaeota, 2 Aenigmarchaeota, 1 Micrarchaeia | 68.8 | 83.9 | 18.9 |
| MBGB-380 | Lokiarchaeia (MBG-B) | 420 | 361 | 360 | none | 99 | 99 Crenarchaeota | 55 | 69.1 | 23.6 |
| MBGB-525 | Lokiarchaeia (MBG-B) | 738 | 670 | 491 | 66 Odin, 37 Heimdall | 142 | 140 Crenarchaeota, 1 Micrarchaeota, 1 uncultured Archaea | 89.8 | 93.3 | 19.2 |

Reference:

Knittel K, Lösekann T, Boetius A, Kort R, Amann R. 2005. Diversity and Distribution of Methanotrophic Archaea at Cold Seeps. Appl Environ Microbiol 71:467-479.
